# Rhizosphere Microbiome and Nutrient Fluxes Reveal Subtle Biosafety Signals in Transgenic Cotton

**DOI:** 10.3390/microorganisms13122702

**Published:** 2025-11-27

**Authors:** Zheng Yang, Yuhang Duan, Renhui Wei, Youlu Yuan, Haoliang Yan, Tong Tang, Haihong Shang

**Affiliations:** 1 Zhengzhou Research Base, State Key Laboratory of Cotton Bio-Breeding and Integrated Utilization, School of Agriculture and Biomanufacturing, Zhengzhou University, Zhengzhou 450001, China; yangz@zzu.edu.cn (Z.Y.);; 2 State Key Laboratory of Cotton Bio-Breeding and Integrated Utilization, Institute of Cotton Research, Chinese Academy of Agricultural Sciences, Anyang 455000, China; 3 Department of Computer Science and Information Technologies, Elviña Campus, University of A Coruña, 15001 A Coruña, Spain

**Keywords:** transgenic cotton, rhizosphere microbiome, biosafety assessment, microbial diversity, nutrient dynamics

## Abstract

Genetically modified crops have transformed agriculture, but their long-term ecological impacts remain incompletely understood. Here we investigate how herbicide-tolerant transgenic cotton affects rhizosphere microbial communities and nutrient cycling over a 28-day growth period using 16S rRNA amplicon sequencing and multivariate analyses. We sampled rhizosphere soil from greenhouse-grown transgenic and wild-type cotton plants at five time points, analyzing microbial diversity, community structure, and nutrient dynamics. Despite initial concerns about transgenic modifications disrupting soil ecosystems, we found no significant differences in microbial α-diversity or β-diversity between genotypes. Only minor, transient changes occurred at the genus level, including <5% shifts in *Flavobacterium* and *Ramlibacter* abundance on day 14, alongside brief nutrient flux variations that normalized by day 28. Notably, transgenic plants showed enhanced above-ground biomass accumulation without compromising rhizosphere stability or soil moisture content. These results demonstrate that herbicide-tolerant cotton maintains rhizosphere homeostasis while improving agronomic performance, supporting the environmental safety of this biotechnology for sustainable agricultural intensification.

## 1. Introduction

The rhizosphere, the soil region immediately surrounding plant roots, provides fundamental support for plant nutrition and health [1,2]. Root exudates comprising sugars, amino acids, organic acids, and secondary metabolites selectively recruit microbial communities, fostering diverse microbiomes that enhance nutrient acquisition, pathogen suppression, and stress tolerance [3,4]. These microbial dynamics are integral to sustainable agriculture, and their disruption may compromise soil fertility and ecosystem service functions [5,6].

Genetically modified crops, engineered for traits such as insect resistance and herbicide tolerance, have fundamentally transformed global food production systems, with cotton (*Gossypium hirsutum* L.) serving as a prominent example [7]. With rapid advances in molecular biology, cotton genetic engineering has expanded from single-trait improvements to regulation of complex physiological processes, including herbicide-tolerant varieties that express the *CP4-EPSPS* gene to confer glyphosate tolerance [8], which have achieved widespread adoption in more than 80 percent of U.S. cotton acreage [9]. Among these advances, herbicide-tolerant cotton has become a major focus, offering effective weed management solutions while raising questions about potential impacts on rhizosphere microbiomes and soil health. These modifications may differentially affect rhizosphere microbiomes by altering root exudate profiles or through herbicide residues that influence microbial communities and nutrient cycling. Herbicide-tolerant varieties particularly facilitate weed management and yield enhancement while reducing chemical inputs [10]. However, their environmental safety assessment primarily depends on whether they exert significant ecological impacts on non-target organisms, including soil microbiomes, particularly given ongoing scientific disagreements regarding long-term ecological risk evaluation [11]. Biosafety assessment of genetically modified crops increasingly focuses on rhizosphere processes, as genetic modifications may alter root exudate composition through changes in plant physiological metabolism, thereby modulating microbial community assembly dynamics. Research findings on the effects of transgenic cotton on rhizosphere microbiomes remain divergent: some studies report no significant changes in bacterial diversity or community structure [12,13], while others observe short-term fluctuations in functional groups associated with nutrient cycling [14]. These discrepancies may arise from methodological differences such as sequencing depth and statistical approaches, variations in cotton genotypes, soil types or climatic conditions across study sites. This inconsistency underscores the necessity for long-term monitoring and multidimensional analyses that integrate community structural dynamics with soil physicochemical factors to distinguish genotype-specific ecological signals from natural fluctuation noise.

High-throughput sequencing technologies have revealed rhizosphere microbiome assembly mechanisms, indicating that cotton rhizospheres are dominated by Proteobacteria, Actinobacteria, and Acidobacteria, with community composition regulated by plant developmental stage, soil chemical properties, and root exudate chemical composition [15]. Genetic modifications may fine-tune these interactions by altering root exudate composition and release, which can selectively influence microbial functional groups driving carbon decomposition, nitrogen fixation and cycling, and phosphorus solubilization [16]. The complexity of rhizosphere nutrient cycling further complicates assessment efforts, as microbially mediated biogeochemical processes including nitrogen mineralization, phosphorus solubilization, and organic matter decomposition form the foundation for maintaining soil health [17]. Although genetic engineering primarily targets specific agronomic traits, it may indirectly disrupt these cycling fluxes by affecting root growth patterns or exudate release, presenting potential risks for soil fertility imbalance. However, empirical data on transgenic cotton’s effects on soil nutrient dynamics remain relatively limited, comprising only a handful of studies that examine factors such as enzyme activities and nutrient availability, often with inconsistent findings. While long-term time-series monitoring is crucial for assessing microbiome stability and distinguishing transgenic effects from natural fluctuations over extended periods—as community dynamics naturally vary with root development and environmental gradients [18]. Initial assessments during early developmental stages can reveal short-term signals that may foreshadow broader impacts. Combining diversity indices with ordination analyses of soil nutrient content provides an effective analytical framework for quantifying these complex dynamics. This approach allows partitioning of β-diversity components [19] and exploration of potential associations between community composition and ecological function.

This study systematically characterizes rhizosphere bacterial communities and their relationships with soil nutrients in herbicide-tolerant transgenic cotton lines during early developmental stages to assess potential ecological and environmental impacts. By focusing on these foundational phases, we aim to identify early indicators of transgenic effects that could inform the design of subsequent long-term monitoring efforts. Using 16S rRNA amplicon sequencing and multivariate statistical approaches, we examine genotype-specific effects on microbial diversity, community structure, and soil-environment correlations and validate ecological stability hypotheses by tracking a series of short-term dynamic signals.

## 2. Materials and Methods

### 2.1. Plant Materials and Cultivation

We obtained transgenic herbicide-tolerant cotton KJC017 (short for transgenic cotton (TG)) and its wild-type control Xinluzao 45 (short for WT) from Cropedit Biotechnology Co., Ltd. (Beijing, China). Plants were grown in polyethylene pots (18 cm diameter, 20 cm height) with seven 1 cm-diameter drainage holes at the greenhouse facility of School of Agriculture and Biomanufacturing, Zhengzhou University, under conditions where each treatment had 6 replicates, each replicate consisted of 4 pots, each pot contained 6 plants, using a soil mixture of substrate soil:perlite = 3:1, and watering and fertilization were consistent across all treatments.

### 2.2. Soil Sampling

We collected above-ground plant material (stems and leaves harvested above the soil surface) and rhizosphere soil at 1, 7, 14, 21, and 28 days. For rhizosphere soil samples, a soil cylindrical cores (8 cm diameter, 8 cm depth) centered on the primary root was first collected. The cotton root system was carefully extracted intact from the soil block, and the soil adhering to the root surface was then gently shaken off and collected as the rhizosphere soil.

### 2.3. Morphological Assessment and Biomass Determination

We photographed above-ground plant parts alongside a meter ruler to document morphological changes. Fresh above-ground biomass was recorded immediately after harvest. Samples were then dried at 80 °C with hourly weighing until mass stabilized to determine dry weight.

### 2.4. Soil Chemical Properties Analysis

Rhizosphere soil subsamples were placed in aluminum containers (46 mm diameter, 25 mm height) and weighed to determine wet mass. Samples were air-dried at room temperature with weighing every 3 days until mass stabilized. Soil moisture content was calculated as: (wet mass − dry mass)/dry mass × 100%. Soil pH was measured potentiometrically in a 1:2.5 (*w*/*v*) soil-water suspension. Organic matter (OM) was determined by the potassium dichromate oxidation method (external heating with oil bath) and titration with ferrous sulfate. Total nitrogen (TN) was measured by the Kjeldahl method, involving digestion with concentrated sulfuric acid and a mixed accelerator, followed by distillation and titration. Total phosphorus (TP) was determined through molybdenum blue colorimetry after digestion, and total potassium (TK) was measured via flame photometry post-acid digestion.

### 2.5. Microbiome Sequencing

We extracted DNA from soil samples using the HiPure Soil DNA Kit (Magen Biotechnology; Guangzhou, China) with five biological replicates per treatment. Sequencing libraries were prepared using the Illumina DNA Prep Kit (Illumina, Beijing, China) and quality-assessed on an ABI StepOnePlus Real-Time PCR System (Thermo Fisher Scientific Inc.; Shanghai, China). Sequencing was performed on a NovaSeq 6000 platform (Beijing, China) using PE250 mode.

### 2.6. Bioinformatics Analysis

Raw sequencing data were processed using FASTP (v.0.18.0) for quality filtering. Clean reads were merged into tags using FLASH (v.1.2.11) with minimum 10 bp overlap and maximum 2% mismatch rate [20,21]. We clustered clean tags into operational taxonomic units (OTUs) at ≥97% similarity using UPARSE algorithm in Usearch (v.11.0.667) [22]. Chimeric sequences were removed using UCHIME before OTU abundance calculations [23].

### 2.7. Taxonomic Annotation

Representative OTU sequences were aligned against SILVA (v.138.2), Greengenes2 (v.2022.10), or UNITE (v.0.0) databases. Taxonomic classification was performed using RDP classifier (v.2.2), and abundance data were visualized using Krona (v.2.6) [24,25,26,27,28].

### 2.8. Alpha Diversity

Alpha diversity was quantified as the Shannon index using the diversity function in the vegan R package (v.2.7-0). Differences in alpha diversity between TG and WT genotypes across time points were assessed using two-way ANOVA followed by Tukey’s honest significant difference post hoc tests (α = 0.05).

### 2.9. Beta Diversity

Beta diversity was calculated as Bray–Curtis dissimilarities on rarefied ASV tables and visualized using principal coordinates analysis (PCoA) with the ordinate function in the phyloseq R package (v.1.50.0). Permutational multivariate analysis of variance (PERMANOVA) was performed on Bray–Curtis distances to test the effects of genotype and time, using the adonis2 function in vegan with 9999 permutations (α = 0.05).

### 2.10. Redundancy Analysis

Redundancy analysis (RDA) was conducted to relate bacterial community composition (Hellinger-transformed ASV abundances) to soil properties (OM, pH, TN, TP, TK), using the rda function in vegan. Environmental variables were standardized, and forward selection (ordistep function) was applied to identify significant predictors (α = 0.05). Radar charts depicted normalized (0–100%) soil property values for each genotype and time point.

### 2.11. Correlation and Mantel Test

Pairwise Pearson correlations among soil properties and between properties and dominant bacterial genera (relative abundances > 1%) were computed using the cor.test function in R (v.4.4.3), with significance adjusted via Benjamini–Hochberg (α = 0.05). Mantel tests assessed correlations between Euclidean distance matrices of soil properties and Bray–Curtis community dissimilarities, implemented with the mantel function in vegan (9999 permutations; α = 0.05). Correlation strengths were visualized as Mantel’s r values, with arrow widths and colors scaled by significance (*p* < 0.01 or 0.01–0.05). All analyses were performed in R.

## 3. Results

### 3.1. Transgenic Cotton Demonstrate Enhanced Above-Ground Biomass Accumulation with Comparable Soil Water Retention

**Figure 1:**
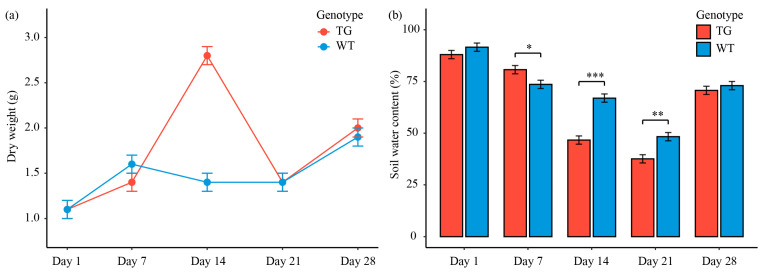
Temporal changes in plant dry weight (**a**) and soil water content (**b**) in the cotton rhizosphere. Line graphs depict mean values (±SE) for transgenic (TG; red) and wild-type (WT; blue) genotypes across days 1, 7, 14, 21, and 28; asterisks indicate significant differences (*p* < 0.05, 0.01, 0.001: *, **, ***).

As illustrated in Figure 1, the above-ground dry weight accumulation trajectory of TG was significantly steeper than that in WT cotton, reaching a significantly higher level on day 14. TG cotton reached its biomass peak on day 14, whereas WT exhibited a minor peak on day 7 and a subsequent decline by day 14. Subsequently, the dry weight of TG began to decline, while that of WT slightly increased but remained lower than that of TG (Figure 1a), indicating that the TG lines may exhibit superior growth performance. Despite the enhanced biomass accumulation, the pattern of soil water content changes was highly similar between the two genotypes, with both TG and WT showing a similar rate of decline over time. Notably, the soil water content in the TG group was generally slightly lower, with a significant decrease only on day 14 (Figure 1b). These results demonstrate that transgenic cotton achieved biomass accumulation while maintaining soil water retention capacity comparable to that of wild-type cotton, indicating improved resource use efficiency without disrupting the fundamental soil–plant water dynamics.

### 3.2. Bacterial α- and β-Diversity Exhibit Temporal Variation with Minimal Genotype Effects

**Figure 2:**
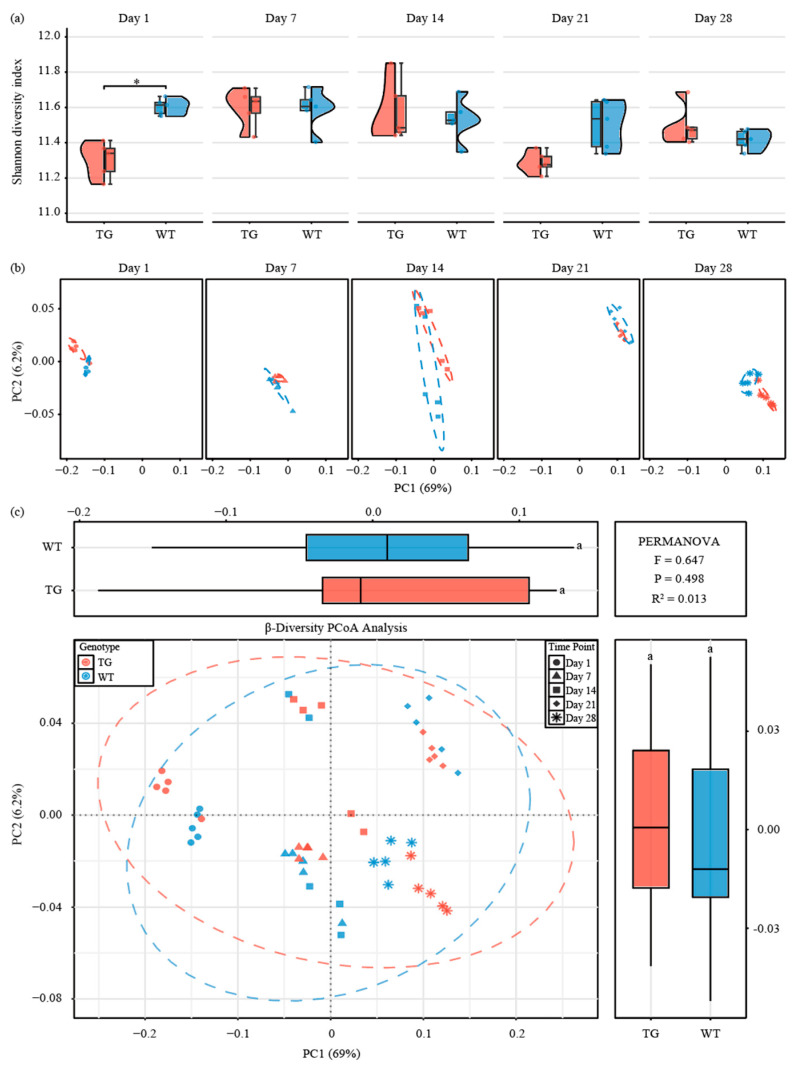
Alpha- and beta-diversity of bacterial communities in the cotton rhizosphere. Box plots show Shannon diversity index for TG (red) and WT (blue) genotypes over days 1–28 (**a**), * *p* < 0.05 versus the WT group; PCoA ordinations illustrate beta-diversity separation by genotype and time (**b**); overall PERMANOVA (**c**) reveals no significant genotype effect (F = 0.647, *p* = 0.498, R^2^ = 0.013). The dotted ellipses represent 95% confidence intervals. Groups marked with the same letter are not significantly different (*p* > 0.05).

To further elucidate the ecological effects of transgenic cotton cultivation, particularly regarding biosafety, we investigated rhizosphere microbiome dynamics, as changes in microbial community structure may signal potential environmental risks. α-diversity remained essentially consistent between TG and WT genotypes across different time points (days 1, 7, 14, 21, and 28), with a significant difference observed only on Day 1 (*p* < 0.05) but no major differences overall, indicating that transgene expression did not broadly impair microbial richness or evenness (Figure 2a).

Principal coordinate analysis (PCoA) of β-diversity at each time points further revealed overlapping distributions of TG and WT samples along PC1 (69% variance) and PC2 (6.2% variance), with the primary variation likely driven by within-group factors such as replicate differences rather than genotype. The overall PCoA ordination showed significant overlap between TG and WT clusters, reaffirming that transgenic cotton did not induce changes in community composition (Figure 2b).

PERMANOVA analysis validated this pattern, with genotype effects being non-significant (F = 0.647, *p* = 0.498, R^2^ = 0.013), indicating that the impact of transgenic cotton on microbial differentiation was minimal and could not be primarily attributed to genotype. Although slight deviations were visible in the β-diversity distance boxplots on days 14 and 21, where TG samples exhibited slightly higher dispersion potentially reflecting transient responses mediated by rhizosphere nutrient fluxes, no statistical differences existed between TG and WT (Figure 2c). This suggests that transgenic cotton did not interfere with microbial diversity.

### 3.3. Genus-Level Composition Exhibits Temporal Dynamics with Minimal Genotype Effect

Analysis of community composition at the genus level revealed that the rhizosphere microbiomes of both TG and WT were dominated by a core set of taxa. Incertae_Sedis consistently had the highest relative abundance across all time points, followed by unclassified sequences. Other genera, including *Acidibacter*, *Dongia*, *Flavobacterium*, *Ramlibacter*, *SM1A02*, *Sphingomonas*, and *Terrimonas*, contributed less.

**Figure 3:**
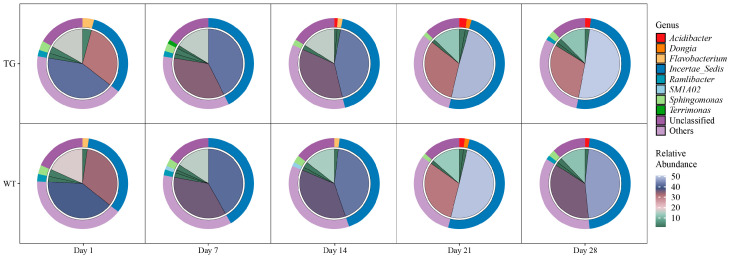
Temporal dynamics of dominant bacterial genera in the cotton rhizosphere. Pie charts represent mean relative abundances (%) for transgenic (TG; **top panel**) and wild-type (WT; **bottom panel**) genotypes at days 1, 7, 14, 21, and 28. Inner slices are color-coded by genus; outer radial gradients scale total abundance.

Community structure changed significantly over time. For example, *Incertae_Sedis* increased from day 1 to day 28, while unclassified taxa decreased, reflecting community maturation associated with root development and nutrient dynamics. Genotype differences were minor: on day 14, Flavobacterium was slightly higher in TG than in WT, while Ramlibacter was slightly lower in TG than in WT, possibly due to biomass-driven exudate changes in the transgenic line. These genres serve distinct ecological functions: *Ramlibacter* contributes to adaptation under oligotrophic conditions through desiccation and starvation tolerance, and participates in cellulose degradation, while *Flavobacterium* is involved in organic matter decomposition and can promote plant growth. However, the observed differences were less than 5% and disappeared by day 21, suggesting that any functional implications were transient and did not affect overall ecosystem stability (Figure 3).

Overall, these findings indicate that transgenic cotton did not compromise community stability or function. Collectively, the data suggests minimal ecological risk of transgenic cotton to the rhizosphere microbiome, supporting its biosafety for agricultural applications.

### 3.4. Temporal Dynamics of Genotype-Modulated Rhizosphere Nutrient Gradients

**Figure 4:**
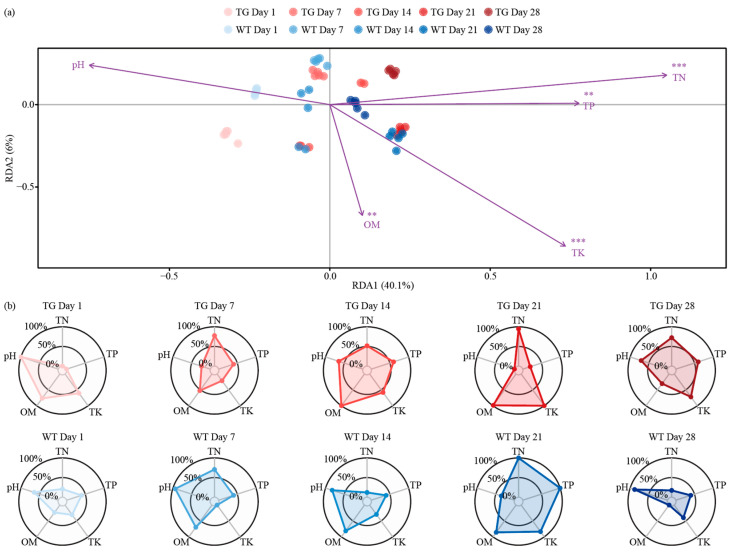
Associations between soil properties and bacterial communities in the cotton rhizosphere. RDA biplot (**a**) positions TG and WT samples by day along RDA1 (40.1%) and RDA2 (6%), with arrows for environmental variables (OM, pH, TN, TP, TK) and significance markers (**, *p* < 0.01; ***, *p* < 0.001); radar charts (**b**) display normalized values of these properties for each genotype and time point. Red polygons represent the TG group, and blue polygons represent the WT group.

The rhizosphere nutrient flux further confirmed the stability of the soil microenvironment in transgenic cotton. Redundancy analysis (RDA) revealed the temporal trajectories of soil properties along RDA1 (40.1% variance) and RDA2 (6% variance). On day 1, samples from WT and TG genotypes clustered tightly in the negative RDA1 region, reflecting initial consistency. Over time (days 7 to 28), samples migrated positively along RDA1, driven by increases in total nitrogen (TN), total phosphorus (TP), and total potassium (TK). In contrast, pH and organic matter (OM) were negatively correlated and gradually decreased over time. On day 14, TG samples slightly deviated from WT but converged by day 28, indicating a transient rather than persistent effect (Figure 4a).

The dynamic patterns of soil nutrient indices for TG and WT over 28 days show that nutrient levels were low and similar for both genotypes on day 1. Subsequently, total nitrogen (TN), total phosphorus (TP), and total potassium (TK) gradually increased, with the most pronounced differences appearing on day 14 and WT were markedly different. However, by days 21–28, the two genotypes converged again, confirming that the impact of transgenic cotton on the soil nutrient environment is transient rather than persistent (Figure 4b).

### 3.5. Microbiome Composition Correlates Moderately with Nutrient Profiles Diversity

**Figure 5:**
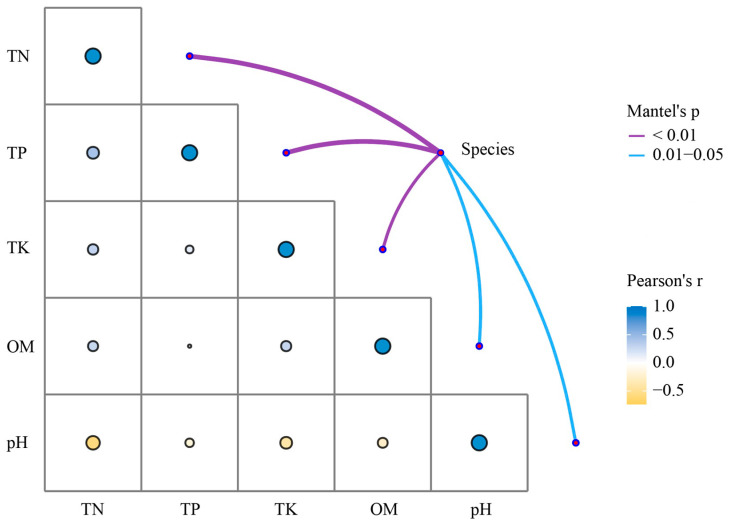
Correlations between soil properties and bacterial species in the cotton rhizosphere. Pairwise comparisons of soil properties are displayed in the lower left, where the color and size of the circles correspond to the direction and magnitude of Pearson’s correlation coefficients (r). The connecting lines indicate the correlation between environmental factors and species, with line widths and colors denoting Mantel’s p (<0.01: purple; 0.01–0.05: blue).

Correlation analysis revealed moderate associations between rhizosphere microbial community composition and soil nutrient profiles, underscoring feedback dynamics in transgenic cotton. Among soil variables, TN and TP exhibited a strong positive correlation, reflecting synergistic nutrient cycling. In contrast, organic matter (OM) and pH displayed moderate negative correlations with TN and TP, consistent with acidification and nutrient mobilization during decomposition. TK showed weak correlations with other nutrients, suggesting a limited role in these interactions. Microbial species composition covaried moderately with the overall nutrient matrix, indicating that community structuring primarily responds to edaphic gradients rather than transgenic modification, as no genotype-specific differences emerged (Figure 5). These patterns delineate a robust nutrient–microbiome network, with subtle perturbations affirming the biosafety of transgenic cotton by preserving soil functional integrity and mitigating fertility risks.

## 4. Discussion

This study demonstrates that herbicide-tolerant transgenic cotton elicits subtle, transient biosafety signals in the rhizosphere, manifested through enhanced above-ground biomass accumulation and minor perturbations in microbial composition and nutrient fluxes, without compromising overall ecosystem stability. While prior investigations into transgenic cotton’s rhizosphere effects have illuminated potential microbial shifts [29,30], inconsistencies arise from methodological constraints, such as limited temporal resolution and omission of integrated nutrient profiling [31,32], which obscure the distinction between genotype-specific impacts and developmental variability. To address these gaps, we employed a controlled greenhouse assay with serial sampling across 28 days post-treatment, coupled with 16S rRNA amplicon sequencing, multivariate ordinations, and correlation analyses, enabling precise delineation of genotype-mediated dynamics in bacterial communities and edaphic properties.

Our findings reveal that transgenic cotton exhibits superior above-ground biomass accrual peaking at day 14, paralleled by comparable soil moisture retention, alongside temporally dynamic bacterial α- and β-diversities that remain statistically indistinguishable from wild-type controls; genus-level compositions show minor, evanescent deviations (e.g., in Flavobacterium and Ramlibacter abundances), while nutrient gradients—marked by transient elevations in total nitrogen, phosphorus, and potassium on day 14—converge over time, with moderate microbiome-nutrient covariation underscoring resilient feedback loops. These observations position the present work within the evolving landscape of rhizosphere ecology [33,34], bridging discrepancies in transgenic crop assessments by emphasizing temporal trajectories and edaphic integrations that prior field-oriented studies on cotton microbiomes have underrepresented [35]. The transient nutrient elevations around day 14 are plausibly linked to early plant–microbe coupling. Peak above-ground biomass and likely increased rhizodeposition may have briefly stimulated copiotrophic bacteria and mineralization, while short-lived shifts in nitrifier–denitrifier balance and heightened plant uptake could re-partition inorganic pools before returning to baseline. Although our current data cannot resolve causality, these mechanisms are consistent with the reversible community changes and nutrient convergence observed.

A key contribution herein is the identification of these fleeting genotype signals as benign indicators of enhanced resource efficiency, rather than harbingers of disruption. Such insights affirm the biosafety of transgenic cotton [36,37], implying that subtle exudate modulations foster adaptive microbial responses without eroding soil functional integrity, thereby bolstering confidence in its deployment for sustainable intensification.

Notably, the novelty of this study lies in its holistic temporal framing of microbiome-nutrient interplay, which extends beyond static snapshots to capture resilience in transgenic systems—a dimension underexplored in Bt-focused biosafety reviews [30,36]. Refining these implications, our results advocate for refined regulatory frameworks that prioritize transient metrics in risk evaluations, with practical applications in optimizing transgenic trait stacking to amplify yield gains while safeguarding rhizosphere services.

This study spans 28 days and therefore emphasizes early-stage rhizosphere responses. Consequently, our findings may not capture longer-term or cumulative effects across full growth cycles and between seasons. We recommend multi-season field trials with repeated sampling at key phenological stages and post-harvest to evaluate persistence, carryover, and interannual variability for robust risk assessment. Moreover, the greenhouse confinement may attenuate field-relevant stressors like climatic variability and multi-trophic interactions, potentially underestimating long-term microbial feedbacks [35]. Although our greenhouse setup enables precise control over variables, it may not replicate the multifaceted interactions in open fields; thus, complementary field trials across diverse agroecological zones are warranted to validate these findings and assess long-term ecological impacts. Additionally, our bacterial-centric analysis overlooks fungal and archaeal contributions [34], alongside functional metagenomics that could elucidate metabolic shifts. This study profiles bacterial diversity using 16S rRNA amplicons; functional potential was not directly measured. Future shotgun metagenomics/metatranscriptomics will enable pathway-resolved assessment of nutrient cycling functions. These limitations delineate avenues for future inquiry, including multi-year field validations to probe persistence. Prospective research should prioritize in situ manipulations of transgenic exudate profiles to dissect causal links with keystone microbial guilds under abiotic perturbations.

## Data Availability

The original data presented in the study are openly available in NCBI SRA under the accession number PRJNA1348340.

## Abbreviations

The following abbreviations are used in this manuscript:

OTU	operational taxonomic unit
PCoA	principal coordinates analysis
PERMANOVA	permutational multivariate analysis of variance
RDA	redundancy analysis
OM	organic matter
TN	total nitrogen
TP	total phosphorus
TK	total potassium
TG	transgenic
WT	wild-type
